# The daily practice of (suspected) coeliac disease management by general practitioners: A qualitative approach

**DOI:** 10.1080/13814788.2018.1516203

**Published:** 2018-10-02

**Authors:** Tom van Gils, Talha G. Senler, Henriëtte E. van der Horst, Chris J.J. Mulder, Gerd Bouma, Henk de Vries

**Affiliations:** a Coeliac Centre Amsterdam, Department of Gastroenterology and Hepatology, Amsterdam UMC, location VU University Medical Centre, Amsterdam, The Netherlands;; b Department of General Practice and Elderly Care Medicine and EMGO Institute for Health and Care Research, Amsterdam UMC, location VU University Medical Centre, Amsterdam, The Netherlands

**Keywords:** Primary care, coeliac disease, qualitative research, follow-up, diagnosis

## Abstract

**Background:** General practitioners (GPs) play a crucial role in diagnosing coeliac disease (CD). However, data on GP management of (suspected) CD patients is sparse.

**Objectives:** To provide insights into the daily practice of diagnosis, treatment, and follow-up of CD by GPs.

**Methods:** A qualitative study using topic list-based semi-structured in-depth interviews with Dutch GPs with more than five years’ experience carried out between January and March 2017. GPs were purposively sampled. The number of GPs interviewed depended on when data saturation was reached. We applied content analysis to the semi-structured interviews.

**Results:** Seven GPs were interviewed, five of whom were female. Analysis of the interviews resulted in three main themes: ‘awareness,’ ‘diagnostics’ and ‘management.’ Vague gastrointestinal symptoms and diarrhoea were often mentioned as a possible presentation of CD. Antibodies were used in CD diagnosis, although some GPs would start a gluten-free diet as a first diagnostic tool. Some GPs diagnosed CD only based on positive antibodies without referring to secondary care or duodenal biopsy analysis. GPs mentioned no role for primary care physicians in the follow-up of CD and noted the important role of dieticians in CD management.

**Conclusion:** The different views of GPs on how to diagnose and monitor CD could be a basis for further research to improve CD detection rate and CD care.

KEY MESSAGES

- Among general practitioners, views vary on how to properly diagnose and manage coeliac.
- The findings of our interviews could function as a basis for further research to improve CD detection and care.

## Introduction

Coeliac disease (CD) is an autoimmune-mediated enteropathy caused by the ingestion of gluten [1]. The prevalence of CD in Western countries is 0.5–1.0% of the general population, of whom the vast majority seem to be undiagnosed [2,3]. The large number of undiagnosed CD individuals is partly caused by a lack of symptoms or the extensive range of symptoms, which are presented by patients. Additionally, symptoms of CD and irritable bowel syndrome (IBS) overlap, which may lead to an under diagnosis of CD once a diagnosis of IBS has been established [4]. Diagnostic work-up of CD includes highly specific and sensitive antibodies—anti-tissue transglutaminase (TGA) and anti-endomysium (EMA) IgA antibodies—combined with duodenal biopsies (showing villous atrophy, crypt hyperplasia and intra-epithelial lymphocytosis in case CD is present (5). A life-long gluten-free diet (GFD) is the only and, at the same time, highly effective and safe treatment for CD patients [5]. Although there is discussion whether detecting CD patients with mild or no symptoms has health-related advantages, there is some evidence that undetected CD can lead to iron deficiency, infertility, osteoporosis and cancer [6–11].

General practitioners (GPs) play a vital role in the Dutch healthcare system since all Dutch citizens contact their GP first if they want to see a medical specialist. Since CD is a clinical chameleon, GPs have to know when to perform CD diagnostic tests [12]. Despite existing CD guidelines, in many patients, a long diagnostic delay [13,14], and sometimes inadequate diagnostic workup is observed [5,15,16]. This includes starting a GFD recommended by GPs based on positive serology only. Additionally, CD is sometimes seen as a disease diagnosed at childhood instead of a disease which could be diagnosed at any age, although the Dutch CD incidence increased between 1995 and 2010 in both adults and children (especially girls) [17].

Scientific data regarding the daily practice of CD management by GPs in case of (suspected) CD are sparse. This study aimed to provide insights into the daily practice of diagnosis, treatment, and follow-up of CD by GPs, using qualitative methods to provide a basis for further research regarding CD in general practice to ultimately improve CD detection rate and CD care.

## Methods

### Study design

For this qualitative study, we used semi-structured in-depth interviews with GPs. The interviews took place in the period between January and March 2017. A medical doctor (HV) with experience in qualitative research trained the two interviewers (TS and TG) to conduct the interviews. We made a topic list to cover all the topics of interest, based on the aim of our study. TG, two CD experts (CM and GB), and a GP (HV) compiled the topic list, inspired by daily practice and current CD guidelines [16,18]. In addition, the Dutch Coeliac Society, a patient advocacy group, reviewed the topic list and the final list was reviewed for completeness by two independent GPs (Supplementary Appendix A). The interviewers avoided closed questions as much as possible and created an atmosphere where the participants could talk freely. Data was collected using a tape recorder from which the records were transcribed verbatim. To check validity, summarized interviews were sent to the participants to check. None of the GPs had comments on these summaries.

### Selection of study subjects

The interview group consisted of practicing GPs. To sample GPs, we asked several GPs from a database of supervisors of primary care interns of students from the VU University Medical Centre for participating in this study. Since we aimed to interview a heterogeneous group, we added several other GPs to the participant group based on their characteristics. We applied purpose sampling based on rural and urban regions, different ages and gender and added GPs with specific interests (IBS and CD) into the group. We estimated a minimum sample size of five GPs and continued until saturation was reached. All GPs had at least five years’ experience.

### Qualitative methods

We used inductive content analysis to organize the qualitative data [19]. While reading the interviews, we wrote open codes in the text. Two authors (TG and TS) coded the manuscript of each interview independently. A third author (HV) checked the codes used. Agreement was reached by discussion. TS, TG, and HV sorted and grouped the codes around central themes and created subthemes. A general description of each theme was ultimately formulated. The parallel process of interviewing and analysing enabled us to observe when saturation of new information was reached. All data were processed and encrypted with Atlas.ti 7.5 software package (Scientific Software Development GmbH).

All participants gave informed consent.

## Results

### Participants

Thirty GPs were approached to take part in the study. Seven GPs were willing to be interviewed. The interviews lasted for 15–30 min. See Table 1 for the GPs’ characteristics.

**Table 1.** Characteristics of participating GPs.

Total number	7
Median age (individual), in years	40 (36, 36, 38, 40, 44, 49, 63)
Gender (males/females), *n*	2/5
Working area (urban/rural), *n*	4/3
Median time of work experience as GP (individual), in years	8 (5, 5, 6, 8, 12, 19, 33)
Specific interest in CD, *n*	1
Specialized in IBS, *n*	1

CD: coeliac disease; GP: general practitioner; IBS: irritable bowel syndrome.

### Themes

The views of GPs on their management of CD were explored. Three main themes were created, each with some subthemes. The main themes and subthemes were:

1. Awareness (prevalence, clinical presentation and groups at risk, self-suspected diagnosis by patients)
2. Diagnostics (additional testing, diagnosis of CD, referring patients)
3. Management (treatment, follow-up, and complications)

#### Awareness

*Prevalence.* All participating GPs rarely diagnosed CD. Only one GP stated that he thinks that 20–30 of his 6500 patients are diagnosed with CD. Some of the GPs mentioned that they frequently tested for CD, but stated that CD-specific blood tests were negative in almost all patients tested.

GP 5: In daily practice, I often perform laboratory tests, TGA. Till now, levels were elevated in only one patient.

*Clinical presentation and groups at risk.* Different clinical presentations of CD were mentioned during the interviews. Abdominal symptoms were most frequently mentioned, of which predominantly vague abdominal symptoms, chronic abdominal pain, and diarrhoea. One GP mentioned pre-existing constipation as clinical presentation of CD. Psychological symptoms such as tiredness were also frequently reported. Other CD clues mentioned were anaemia (in combination with vague abdominal symptoms), flatulence, and aphthous stomatitis. Several times, GPs mentioned that it is difficult to distinguish CD and IBS based on a similar clinical presentation.

Most participants recalled that CD is a disease most often diagnosed during childhood, although asymptomatic or mild symptoms in adult CD were also mentioned by some GPs. Child-specific symptoms recalled by the participating GPs were a failure to thrive, bulging belly, grumpy children, and symptoms starting after switching from breast milk to normal food.

GP 3: Basically, real coeliac disease, the heavy ones, starts most often really young. Sometimes, I refer older patients with IBS-like complaints [to secondary care] resulting in lactose intolerance or coeliac disease diagnosis.

I: And are there certain groups in which you consider it [CD] more often, so groups at risk?

GP 4: No, no, I don’t think so.

I: Or certain age groups where you would expect it more often?

GP 4: I don’t consider it in elderly patients, more often in young adults.

Several GPs mentioned specific groups of patients who have an increased risk for developing CD, especially IBS-like patients, as well as a family member with CD.

*Self-suspected diagnosis by patients.* All participants reported that some of their patients without a previous diagnosis of CD claimed to have symptoms after consuming gluten-containing food and some reported that ‘gluten allergy’ or a GFD have become a hype. The diagnostic problem of diagnosing CD in patients who are on a self-prescribed GFD was also mentioned.

GP 4: I do not know how to do that [CD testing under a gluten-free diet], because the antibodies disappear, isn’t it. But not after two weeks, that is what I expect.

#### Diagnostics

*Additional testing.* GPs frequently mentioned that there is a broad differential diagnosis in cases where CD is suspected. For the participating GPs, serological testing for CD was the first diagnostic step, although only some knew which exact test he or she usually uses. Low threshold testing was reported and motivated by the wide range of symptoms patients present with, the low costs of the serological tests and the requests to test by the patients.

Opinions about when to perform serological tests in patients who are on a GFD varied widely: only testing when patients were on a gluten-containing diet, not knowing whether it is possible to use serological tests when a patient is on a GFD and first waiting for the clinical effects of a GFD before performing any test. Only a GP interested in CD was aware of the existence of HLA-DQ testing. None of the GPs had ever seen a patient who had used a CD point of care test at home.

GP 4: Some people tell me that they experience more symptoms consuming bread. In these cases, I ask them to stop eating bread and to look at what happens, whether there will be some improvement.

I: And if the patient stops eating bread, what will you do next?

GP 4: Blood analysis.

I: Still removing bread from the diet?

GP 4: Yes, but not too long… otherwise the test won’t give a result [positive result if the patient has CD].

GP 6: Well, it [CD antibodies] is positive, so it is a gluten allergy. These patients will be on a gluten-free diet and just see whether they will improve. I would recommend discussing this [GFD] with a dietitian.

*Diagnosis of coeliac disease.* Different opinions regarding how to diagnose CD using antibodies were present, varying from a positive TGA as confirmation for CD diagnosis to duodenal biopsies after positive TGA necessary for CD diagnosis. According to all GPs, CD was excluded when serology was negative.

*Referring patients*. In case of CD suspicion, in most cases based on positive serological tests, GPs referred patients to a gastroenterologist, internal medicine department or a (gastrointestinal interested) paediatrician. Other steps taken after identification of positive antibodies were immediately starting a GFD, and after that referring to secondary care, or only referring when symptoms persist, and referring to a general or CD specialist dietician instead of secondary care. The reason for referral to a dietician instead of a physician was that dieticians have more knowledge about food than a gastroenterologist does.

I: So do you advise to start a gluten-free diet, only with [positive CD] antibodies?

GP 3: Yes, but in general, I still refer to a paediatrician. These days they do not often perform an endoscopy or biopsy anymore, isn’t it?

I: … and if it [CD antibodies] is positive, do you refer to a medical specialist or…?

GP2: No, I know too little about this. I do not know so much about food, and I am very pleased that others know more about it, so that will be the next step.

I: So the next step is referring to a dietician? Or a gastroenterologist?…

GP2: A dietician, and if the result is lacking, then to a gastroenterologist, yes.

I: So a biopsy is not necessary?

GP2: That is what I think.

I: In summary, is it true that you refer [a patient with positive CD antibodies] to a dietician instead of a gastroenterologist or internist because a dietician knows more about food?

GP2: Yes, that is true; I think a dietician knows more about food than a gastroenterologist.

Although participating GPs stated that in case of negative serology CD was excluded, they would ultimately refer patients with persisting (gastrointestinal) symptoms, or patients who would like to be referred, to secondary care.

#### Management

*Treatment.* All participating GPs were aware that the treatment of CD is a GFD and stated that they are no experts on GFDs. One GP stated that a small amount of gluten should not be a problem for CD patients. Another GP mentioned that he could not imagine that CD patients without symptoms would consume a GFD.

GP 4: …So a patient with CD eats some gluten without any symptoms. This patient will start eating some gluten again, isn’t it?

*Follow-up and complications.* We observed a wide range of opinions regarding the follow-up of CD patients: follow-up by a medical specialist, GP or dietician. It has been mentioned that it is unclear who has to perform follow-up and what to do during follow-up, with one GP who was willing to perform follow-up with recommendations by a medical specialist. All GPs referred to a dietitian for food-related education and questions from patients.

Several GPs responded that they were not aware of any complication related to CD. Osteoporosis, vitamin deficiency, anaemia, failure to thrive, intellectual disability and aphthous stomatitis were mentioned as possible complications of CD. One GP mentioned that one of his patients was afraid of developing an enteropathy-associated T-cell lymphoma and one GP mentioned that training regarding complications in CD would be useful for daily general practice.

I: When you think about coeliac disease, the disease itself, do you think about certain complications?

GP 4: Only the vitamin deficiencies, that’s it.

## Discussion

### Main findings

The main findings based on our in-depth interviews with seven GPs were:

1. *Awareness*: CD suspicion is most common in patients with abdominal discomfort and other gastrointestinal symptoms.
2. *Diagnostics*: The importance of serological testing is known by GPs. Low threshold testing was reported and motivated by the wide range of symptoms patients present with, the low costs of the serological tests and the requests to test by patients. Not all GPs referred patients to a medical specialist once antibodies were positive.
3. *Management*: There was a wide variety of opinions on who has to perform follow-up. All GPs stated an essential role for dieticians in the management of CD patients.

### Strengths and limitations

The most important strength of this study was that this study is the first study exploring the daily practice of CD management by GPs. This knowledge could be a basis for further research on the awareness and management of CD, which could ultimately provide evidence for future CD guidelines including the role for primary, secondary and tertiary caregivers in the management of CD. However, both larger quantitative and more specific, in-depth qualitative studies are needed.

A limitation could be that GPs may have read the CD guidelines before the interview, resulting in answers based on guidelines rather than daily clinical practice.

### Interpretation of the study results in relation to existing literature

*Awareness.* Based on our data, most GPs have insight into the variety of clinical presentations of CD including a combination of symptoms such as ‘tiredness and abdominal symptoms’ or ‘anaemia and chronic abdominal symptoms,’ which is in line with the recommendations of the Dutch GP guidelines (NHG guidelines) [18]. However, knowledge regarding age at the time of CD diagnosis, with a predominance of presentations above the age of 18 [20–22], and the risk of complications in adult diagnosed CD patients need more attention by GPs. This knowledge should lead to a lower threshold to test adults for CD in the presence of clinical suspicion.

*Diagnostics.* All interviewed GPs knew that a blood test for CD is available. After the identification of TGA as CD-specific and sensitive auto-antibody in 1997 [23], testing patients for suspicion for CD has become much easier. Over the last few years, this has led to an increase in patients diagnosed with CD by GPs [24]. A recent UK study showed that approximately 80% of the GPs often or always checked CD serology in patients with symptoms suggestive for IBS and only 2% never performed these test in IBS suspected patients [25]. There is considerable variability in the kind of antibodies requested by GPs [26], but data regarding Dutch GPs are lacking. Despite the very sensitive and specific CD antibodies, referring patients with positive CD serological tests to a gastroenterologist or a paediatrician interested in gastroenterology is still recommended since histological analysis of duodenal biopsies is the gold standard with well-described exceptions in children [18,27,28]. Referral rates may dramatically improve when a specific laboratory comment is attached to positive antibodies including the advice to refer the patient to a gastroenterologist [29]. An inaccurate diagnostic workup could lead to the prescription of an unnecessary, lifelong, expensive and strict GFD, which is undesirable. A GFD without any medical need is also often consumed by self-reported gluten or wheat sensitive individuals, an entity with unknown pathophysiology in the absence of CD and without any currently known specific biomarker with a prevalence of 6% and 13% respectively in the Dutch and UK general population [30,31]. In these individuals, a proper diagnostic workup for CD is required including a gluten rechallenge [32]. When a patient does not want to be rechallenged, HLA-DQ typing could be useful since the absence of HLA-DQ2 and HLA-DQ8 exclude the presence of CD.

*Management.* Once CD is diagnosed, the treatment consists of a strict GFD after which symptoms in the vast majority of patients resolve. It has been shown that even asymptomatic CD patients have benefit of a GFD, although only one randomized controlled trial in relatives of CD patients has been conducted [11,33]. Therefore, referring all newly diagnosed CD patients to a dietician is essential to ensure a strict GFD is followed. The benefit of a GFD is also supported by the observation that visiting primary care medicine after the diagnosis of CD has been dropped with approximately 40%, which could be explained by the resolving of symptoms after starting the diet [34]. After CD is diagnosed, less is known about how to perform follow-up. The Dutch GP guidelines recommend a follow-up of CD in secondary care [18]. If a patient is not willing to visit secondary care for follow-up or is not willing to consume a GFD, the Dutch GP guidelines recommend to follow-up amongst others weight and TGA yearly [18].

### Implications for clinical practice

This study aimed to create a basis for further research into the daily practice of diagnosis, treatment and follow-up of CD by GPs to ultimately improve CD detection rate and CD care. In this study, we showed that the views of GPs regarding management of CD vary widely and that the recommendations of guidelines are not always followed. Discordance exists in how to diagnose CD correctly and how to follow-up patients with CD. Attention points for clinical GP practice in diagnosing and treating CD patients, based on comparing the interviews and current guidelines and literature, are summarized in Box 1.

Box 1 Attention points

Clinical presentation

- Coeliac disease is most frequently diagnosed in adults
- General practitioners should be aware of different groups at risk for coeliac disease: first degree family members of coeliac disease patients, Down’s syndrome, diabetes mellitus type I, thyroid disease
- Coeliac disease is a clinical chameleon with i.e. iron deficiency, infertility, osteoporosis and weight loss as presentations beside gastrointestinal symptoms

Additional testing

- Coeliac disease specific antibody tests and duodenal biopsies have to be performed under a gluten-containing diet
- HLA-DQ typing could be used to exclude coeliac disease, even when patients follow a self-initiated gluten-free diet
- Diagnostic work-up differs between children and adults. In certain circumstances, duodenal biopsies are not needed in children whereas duodenal biopsies are mandatory in adults

Treatment

- The treatment of all coeliac disease patients is a strict gluten-free diet. A gluten-restricted diet has no place in the treatment of coeliac disease patients

## Conclusion

In this study, we observed different views regarding how to diagnose and follow-up CD patients by GPs, although the management of CD, a GFD, was known by the interviewed GPs. This research could be a basis for further research to improve CD detection rate and CD care.

## Supplementary Material

Supplementary Material
