# Supplementary material for: The daily practice of (suspected) coeliac disease management by general practitioners: A qualitative approach
Source: Eur J Gen Pract. 2018 Oct 2;24(1):236–42. doi: 10.1080/13814788.2018.1516203 (PMC6171459; doi:10.1080/13814788.2018.1516203)
Supplement: Supplementary Material [file IGEN_A_1516203_SM1198.docx]

# Appendix A

***Item List interview ‘management of GPs in (suspected) coeliac disease’ (original in Dutch)***

**Diagnostics**

1. What are your experiences in diagnosing coeliac disease?
2. When do you suspect coeliac disease?
3. In the presence of which symptoms do you consider a coeliac disease?
4. Which patient groups do you consider to screen for coeliac disease? (for example a positive family history?)
5. What do you do when a coeliac disease is suspected?
6. What do you ask to test your hypothesis?
7. Which physical examination do you perform?
8. Which additional tests do you perform?
   1. Which kind of blood test do you perform? (by asking questions about (different types of) antibodies, HLA-DQ),
   2. Coeliac disease
   3. ?
   4. Do you advice to try a gluten-free diet without additional testing?
   5. Are there patients who performed a point of care (home) test by themselves?
9. Do you have problems or unusual situations regarding the diagnosis of coeliac disease?
10. Is there a difference for you between diagnosis in children and adults, and if so, what differences?
11. How do you deal with people asking for additional testing when they do not belong to a risk group or do not have symptoms?
12. How do you deal with patients who have tested negative coeliac disease antibodies, but where you still clinically suspect coeliac disease?
13. How are you dealing with diagnostic tests in patients who are already on a gluten-free diet in case you or the patients suspect the presence of coeliac disease? (by asking questions about reintroduction of gluten, duration of reintroduction, etc.)
14. When is the diagnosis of coeliac disease excluded?

**Referring**

1. When do you refer a patient with suspected coeliac disease?
   - 1. To which specialist (s) do you refer?
     2. How is your experience regarding the cooperation with these specialist(s)?
     3. Do you consult a (coeliac disease) specialist(s)?

**Follow-up**

1. What is your role in the follow-up of patients with coeliac disease?
2. Do you refer to a dietician?
3. Do you refer to a dietitian who is specialized in coeliac disease?
4. What is your experience with referring to a dietician?
5. Of which complications in coeliac disease patients are you aware?
6. When would you refer patients for specific complications and to whom?
